# Post-transplant glomerular diseases: update on pathophysiology, risk factors and management strategies

**DOI:** 10.1093/ckj/sfae320

**Published:** 2024-10-24

**Authors:** Anna Regalia, Matteo Abinti, Carlo Maria Alfieri, Mariarosaria Campise, Simona Verdesca, Francesca Zanoni, Giuseppe Castellano

**Affiliations:** Department of Nephrology, Dialysis and Renal Transplantation, Fondazione IRCCS Ca’ Granda Ospedale Maggiore Policlinico, Milan, Italy; Department of Nephrology, Dialysis and Renal Transplantation, Fondazione IRCCS Ca’ Granda Ospedale Maggiore Policlinico, Milan, Italy; Department of Clinical Sciences and Community Health, Università degli Studi di Milano, Milan, Italy; Post-Graduate School of Specialization in Nephrology, University of Milan, Milan, Italy; Department of Nephrology, Dialysis and Renal Transplantation, Fondazione IRCCS Ca’ Granda Ospedale Maggiore Policlinico, Milan, Italy; Department of Clinical Sciences and Community Health, Università degli Studi di Milano, Milan, Italy; Department of Nephrology, Dialysis and Renal Transplantation, Fondazione IRCCS Ca’ Granda Ospedale Maggiore Policlinico, Milan, Italy; Department of Nephrology, Dialysis and Renal Transplantation, Fondazione IRCCS Ca’ Granda Ospedale Maggiore Policlinico, Milan, Italy; Department of Nephrology, Dialysis and Renal Transplantation, Fondazione IRCCS Ca’ Granda Ospedale Maggiore Policlinico, Milan, Italy; Department of Nephrology, Dialysis and Renal Transplantation, Fondazione IRCCS Ca’ Granda Ospedale Maggiore Policlinico, Milan, Italy; Department of Clinical Sciences and Community Health, Università degli Studi di Milano, Milan, Italy

**Keywords:** de novo glomerular disease, kidney transplantation, primary glomerulonephritis, recurrent disease

## Abstract

In recent years, advancements in immunosuppressive medications and post-transplant management have led to a significant decrease in acute rejection rates in renal allografts and consequent improvement in short-term graft survival. In contrast, recent data have shown an increased incidence of post-transplant glomerular diseases, which currently represent a leading cause of allograft loss. Although pathogenesis is not fully understood, growing evidence supports the role of inherited and immunological factors and has identified potential pre- and post-transplant predictors. In this review, we illustrate recent advancements in the pathogenesis of post-transplant glomerular disease and the role of risk factors and immunological triggers. In addition, we discuss potential prevention and management strategies.

## INTRODUCTION

Kidney transplantation (KTx) is the best renal replacement therapy, providing better survival and quality of life compared with dialysis [1, 2]. Improvements in immunosuppressive medications, donor–recipient matching and post-transplant management have significantly increased short-term graft survival, whereas long-term graft survival rates are still impacted by complications [3]. In recent years, post-transplant glomerular diseases (GDs), including recurrence of primary glomerular disease (pGD), de novo GDs and donor-derived GDs, have emerged as a significant cause of graft failure and currently represent the third-leading cause of allograft loss [4, 5].

Individuals with pGDs, being young and otherwise healthy, are ideal KTx candidates. Indeed, several registries have shown that pGD is a frequent cause of native kidney failure among KTx recipients, ranging from 18% to 30% in Europe and the USA [5–8, 9].

Recurrence of native pGD is the most common form of post-KTx GD. Registry data have shown that the overall post-KTx pGD recurrence rate is 10–20%, causing graft loss in up to 50% of cases [5, 7, 8, 10]. These rates are often impacted by the duration of follow-up in observational studies as well as kidney biopsy policy differences across many centres. Each pGD diagnosis has a different recurrence rate, impact and consequences on graft survival [8, 11, 12].

De novo GDs are defined as GDs that occur post-transplantation in recipients not previously affected by a GD in their native kidneys. They represent the second most common cause of GD in KTx recipients, although their epidemiology and pathogenesis are only poorly understood.

Despite recent advancements, it is still not clear how to prevent, early recognize and treat both recurrent pGDs and de novo GDs in KTx recipients. In this review we aimed to elucidate current knowledge on the epidemiology, pathogenesis, predictors and prognostic factors of recurrent and de novo GDs after transplantation in their most common forms, namely focal segmental glomerulosclerosis (FSGS), immunoglobulin A nephropathy (IgAN), membranous nephropathy (MN) and membranoproliferative glomerulonephritis (immune complex mediated and complement mediated), and to propose practical clinical algorithms for their management.

## PRIMARY FSGS (pFSGS)

### Definition, epidemiology and pathogenesis

FSGS has an estimated and constantly increasing incidence of 10 cases per million worldwide and has become the most frequent GD causing kidney failure [13]. It can occur at any age, accounting for 20–30% of all cases of nephrotic syndrome in adults [12, 13].

FSGS has a non-specific histological pattern that identifies podocytopathies of different aetiologies, including maladaptive, virus-associated, drug-induced (secondary), genetic and idiopathic (primary) forms [13]. A proper distinction among these forms is crucial for post-transplant management, since pFSGS has a high rate of recurrence while other forms much rarely recur [13, 14].

The Kidney Disease: Improving Global Outcomes (KDIGO) 2021 guidelines suggest genetic testing in children and young adults with a clinical course consistent with genetic FSGS to determine the risk of recurrence [15–17]. FSGS recurrence risk is virtually zero in all genetic FSGS forms except for those caused by podocin mutations. In addition to informing regarding recurrence risk, genetic testing allows for a better selection of appropriate living donors and the avoidance of ineffective and potentially harmful therapies. To date, genetic sequencing panels have identified Mendelian mutations in up to 30% of patients <25 years of age and 12% of adults with steroid-resistant nephrotic syndrome [15, 16].

Although the pathogenesis of native and recurrent pFSGS remains largely unknown, it is thought it involves the presence of one or more circulating factors that affect podocyte and glomerular permeability. Several studies have focused on the identification of candidate permeability factors. These include apolipoprotein A-1 (ApoA1), soluble urokinase-type plasminogen activator receptor (suPAR), cardiotrophin-like cytokine factor 1 (CLCF1), plasminogen activator inhibitor type-1 (PAI-1), angiotensin II type 1 receptors (AT1Rs), dystroglycan (DG), microRNAs, metalloproteinases (MMPs), forkhead box P3 (FOXP3) and poly [ADP-ribose] polymerase 1 (PARP1) [18]. However, to date, none of these molecules have proven to be involved in the pathogenesis of pFSGS. Recent data have proposed anti-nephrin antibodies as a candidate soluble factor in primary podocytopathies [19, 20]. In a large-scale multicentre study involving >500 individuals with nephrotic GDs, anti-nephrin antibodies were detected in 46 of 105 (44%) adults with minimal change disease, in 94 of 182 (52%) children with idiopathic nephrotic syndrome and in 7 of 74 (9%) adults with pFSGS. The study showed a correlation with disease activity, suggesting a potential pathogenic role, although further research is required to confirm this finding [20]. Recently, pre-transplant anti-nephrin antibodies have been associated with recurrent pFSGS [21, 22].

Genome-wide association studies (GWASs) have helped elucidate the pathogenesis of native primary podocytopathies, including pFSGS. Common variants within the major histocompatibility complex (MHC) locus, *NPHS1*, a gene encoding for nephrin, and other genes involved in the regulation of T and B lymphocytes, monocytes and eosinophil activity are associated with increased susceptibility to paediatric steroid-sensitive nephrotic syndrome [23, 24]. These findings further suggest that primary podocytopathies are immune-mediated GDs and support the role of immune-activating circulating factors [25]. Given its rarity, no GWAS of post-KTx pFSGS recurrence has been published to date.

Observational data have shown that the pFSGS recurrence rate after the first transplant significantly varies among studies (from 10% to 55%), increases in following transplants (up to 80–100%) and is associated with an increased risk of graft loss [17]. However, a thorough evaluation of potential secondary forms, which requires extensive clinical, biochemical and histological [especially electron microscopy (EM)] data, is often missing in retrospective studies and registries. This may result in erroneous misclassification of secondary forms of FSGS into pFSGS and the consequent underestimation of pFSGS recurrence rates.

In an effort to combine detailed observational data across many centres, a recent large-scale study involving 11 742 KTx recipients [Post-TrANsplant GlOmerular Disease (TANGO)] explored the epidemiology of pGD recurrence after transplant [26]. Among the 176 KTx recipients with biopsy-proven pFSGS, 57 (32%) experienced disease recurrence at a median time of 1.5 months after transplant and 39% of them experienced graft loss over a median of 5 years [27].

### Clinical presentation and histopathology

Classically, pFSGS in native kidneys is associated with progressive nephrotic syndrome [12]. After transplant, in most cases recurrence occurs early (within a few hours to 1–2 weeks post-transplant), further supporting the role of circulating permeability factors in podocyte damage [28]. Patients with early recurrence generally develop massive proteinuria, whereas late recurrence is commonly characterized by a slow development of nephrotic syndrome within months or years [29].

In early transplant biopsies, classic sclerosant lesions might not still be present and diagnosis relies on the demonstration of diffuse podocyte foot process effacement by EM [30]. The typical histological lesions observed by optical microscopy seem to appear around 2 weeks after transplant [31]. Notably, the histological subtype of recurrent pFSGS based on the Columbia classification may differ from that observed in native kidneys [31]. In advanced histologic stages, it can be difficult to distinguish pFSGS recurrence from secondary FSGS forms caused by calcineurin inhibitor (CNI) toxicity, obesity or hypertension.

### Recurrence risk and prognostic factors

Factors associated with recurrence of pFSGS are mainly derived from small retrospective studies and registry data, affected by small power and selection bias due to variable omittance. They include prior graft failure due to recurrence, nephrotic syndrome at presentation, rapid FSGS progression in native kidneys, younger age, White race, nephrectomy of native kidneys and living related donor transplant [27, 32, 33].

Regarding histopathology, a small study showed that collapsing FSGS was associated with a lower likelihood of recurrence, although this finding might be biased by the incomplete exclusion of secondary forms [34]. In addition, Monzumi *et al.* [35] proposed that native forms with mesangial proliferation have a higher recurrence risk, reflecting a more severe disease activity.

Few studies have investigated the role of circulating antibodies in predicting recurrence risk. A panel of seven antibodies listed by Delville *et al.* [32] (CD40, PTPRO, CGB5, FAS, P2RY11, SNRPB2 and APOL2) showed potential associations with recurrent FSGS but were never further validated.

Two studies have recently demonstrated the role of pre-transplant anti-nephrin antibodies in predicting FSGS recurrence risk with high specificity [21, 22]. In addition, in recurrent cases with circulating anti-nephrin antibodies, allograft biopsies have shown co-localized glomerular deposition of nephrin and immunoglobulin G (IgG), suggesting a potential pathogenic effect, although it requires further validation [21].

In light of increasing evidence for a pathogenic role of autoimmunity, also supported by GWASs in native pFSGS, future research should focus on the assessment of donor and recipient genetic susceptibility factors, especially within the human leucocyte antigen (HLA) region, recipient autoantibodies and their interactions.

Failure to respond to treatment appears the main negative prognostic factor in recurrent pFSGS, as shown in recent data from the TANGO study, where graft loss was mainly confined to patients who failed to enter remission [20 of 31 patients (65%)] [27].

### Recurrence prevention

The KDIGO 2020 guidelines suggest avoiding routine use of pre-transplant plasma exchange (PEX) or rituximab (RTX) to reduce the risk of recurrence [36]. In fact, previous literature has never found a significant effect of prophylactic PEX on pFSGS recurrence, but many limitations due to small sample size, retrospective analyses and no randomization should be considered [37]. Kwon *et al.* [38] recently found that pre-transplant treatment with PEX was associated with a lower risk of recurrence in 99 patients with kidney failure due to pFSGS. Data on prophylactic use of RTX are even more limited [37, 39]. Overall, according to a recent meta-analysis, the use of RTX with or without PEX, or PEX alone, is not associated with a lower risk of pFSGS recurrence [40]. Regarding post-transplant pFSGS recurrence prevention strategies, data are scarce and contradicting [41, 42]. To date, few clinical trials have assessed the pre-emptive use of RTX, bleselumab (anti-CD40 monoclonal antibody) or adrenocorticotropic hormone (ACTH) in KTx recipients with native pFSGS (NCT03763643, NCT02921789 and NCT02683889, respectively) [43].

### Recurrence management

To date, targeted therapies for pFSGS recurrence are still lacking and treatment strategies have not changed significantly over the last decades [44]. Daily proteinuria measurements are advised from the first day after KTx to promptly recognize early disease recurrence [37].

RTX and PEX are the most used treatments of pFSGS recurrence (80% of cases) [39]. RTX acts on podocytes directly or by immune B cell depletion, while PEX is supposed to remove the pathogenetic circulating factors. Some centres prefer immunoadsorption instead of PEX, due to the more selective capacity to remove immunoglobulins, the preservation of coagulation factors and the absence of substitution fluids. However, immunoadsorption is more expensive and less available, and its clinical superiority has never been clearly demonstrated [45]. Both techniques appear to effectively reduce proteinuria, even if it is not really known which circulating factors are being removed [46]. The use of different therapeutic strategies and outcome definitions explain the wide variability of remission rates reported in the literature [39]. Generally, partial or complete remission occurs in up to 60% of patients and is associated with better graft survival [27]. Lanaret *et al.* [47] evaluated the use of RTX in addition to PEX, corticosteroids and CNI [standard of care (SOC)] in KTx recipients with pFSGS recurrence. This multitarget approach was found to ameliorate remission (achieved in 80% of cases) and long-term prognosis, with graft survival at 10 years after recurrence >50%. In addition, the use of RTX in case of SOC failure or in early PEX discontinuation was able to maintain remission without increasing the risk of infection. Intravenous cyclosporine is another common treatment for pFSGS recurrence, since it stabilizes the podocyte cytoskeleton acting on synaptopodin, but nephrotoxicity and logistical efforts related to continuing infusions limit its use [48].

In PEX- or RTX-resistant cases, other pharmacological strategies have been used, but they mainly refer to case reports. They rely on emerging therapies used in native kidneys to target specific pathogenic pathways [44]. Among these, ACTH reduces foot process effacement and podocyte apoptosis, leading to disease remission in animal FSGS models [49, 50]. Low-density lipoprotein apheresis has been shown to reduce proteinuria through improved intracellular drug transport with low lipotoxic effects [44]. Other anecdotical reports consider the use of abatacept (CTLA4-IgG) [51, 52] and ofatumumab (fully human anti-CD20 monoclonal antibody) [53, 54]. To date, no study has specifically evaluated the effect of sparsentan (dual endothelin and angiotensin receptor blocker) on recurrent pFSGS treatment.

**Figure 1:**
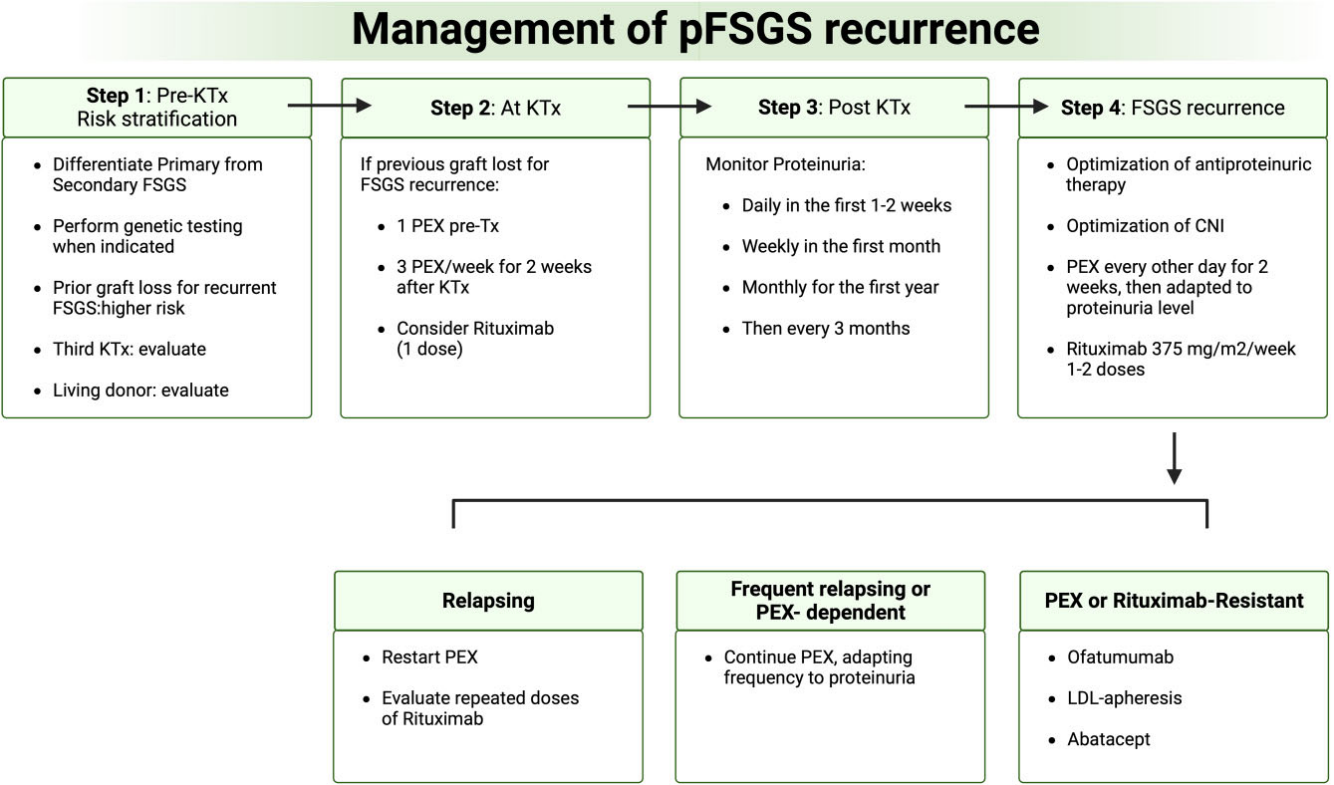
Algorithm for pFSGS recurrence in KTx. LDL: low-density lipoprotein.

Figure 1 summarizes management strategies of pFSGS recurrence.

In summary, the identification of true pFSGS forms is critical for the selection of individuals who are likely to benefit from pre-emptive treatments, surveillance strategies and immunosuppressive therapies. Future studies aimed at identifying pathogenic circulating permeability factors will help develop targeted therapeutic strategies to improve kidney outcomes in both native and recurrent pFSGS.

## PRIMARY IGAN

### Definition, epidemiology and pathogenesis

IgAN is the most frequent form of pGD [55] and is responsible for kidney failure in up to 40% of cases >20 years of age [56]. Considering that patients affected by primary IgAN are generally young and affected by few comorbidities, KTx is the treatment of choice when kidney failure occurs [57].

Primary IgAN recurrence after KTx is a time-dependent phenomenon, since it is rarely reported before the third year after KTx [8, 58]. The recurrence rate appears to be strongly influenced by the biopsy policy of the centre, ranging from 10 to 30% in indication biopsies and 25 to 53% in protocol biopsies [32]. Recurrence does not appear to affect short-term graft survival, but in studies with longer follow-up it is associated with a higher risk of graft loss [39]. According to a recent study, disease recurrence occurred in 82 of 504 KTx recipients with IgAN, with a cumulative incidence of 23% at 15 years. The median time to recurrence was 3.4 years, leading to graft loss in nearly 44% of cases [59].

IgAN is a polygenic immune-mediated GD. Recent large-scale GWASs have found that numerous HLA and non-HLA loci are associated with increased IgAN risk [60–63]. These genetic variants are involved in complement cascade, innate defence against mucosal infections, antigen presentation and to the overproduction of galactose-deficient IgA1 (Gd-IgA1), which is thought to be the first of a multihit pathogenic model [55, 61, 64, 65]. This leads to the formation of anti-Gd-IgA1 IgG antibodies (second hit), circulating immune complexes and macro-aggregates of polymeric Gd-IgA1 that can deposit in kidneys or create *in situ* complexes (third hit) that cause mesangial proliferation, cytokine synthesis and complement activation (fourth hit) [66–68].

A polygenic risk score (PRS) derived from the largest GWAS published to date was associated with earlier disease onset and higher risk of kidney failure, suggesting that higher genetic susceptibility may be predictive of a more aggressive disease [65]. Even if less is known about IgAN recurrence pathogenesis, and GWAS approaches in this population may be underpowered, it has been hypothesized that similar pathogenetic mechanisms are involved. For this reason, the application of known native IgAN genetic risk (and PRS) in the transplant setting may help elucidate the pathogenesis and related risks of IgAN recurrence.

### Clinical presentation and histopathology

Clinical manifestations of IgAN recurrence mimic the primary forms in native kidneys. Notably, microscopic haematuria can be absent in up to one-quarter of patients as reported by Moroni *et al.* [69]. Regarding the histological presentation, Oxford classification criteria based on the MEST-C score have been successfully applied to IgAN recurrence and can provide useful prognostic information for graft survival [70–72].

It is important to differentiate ‘clinical’ from ‘histological-only’ IgAN recurrence. Through protocol biopsies, Ortiz *et al.* [73] identified the presence of mesangial IgA deposition in the absence of clinical manifestations in up to 33% of patients with IgAN 2 years after KTx. In some cases, IgA deposits disappeared in subsequent biopsies. Some studies have demonstrated that histological-only IgAN may originate from apparently normal donors that have latent IgA deposits in the kidney [35]. Nevertheless, when kidneys with latent mesangial IgA deposits were transplanted in non-IgAN patients, the IgA was rapidly cleared [59].

### Recurrence risk and prognostic factors

Clinical factors associated with a higher risk of IgAN recurrence have been mostly addressed in relatively small studies and registries. They include a rapidly progressive course of disease in native kidneys, crescents on the native kidney biopsy [74], male gender, younger age at transplantation [73], early steroid withdrawal (ESW) [75], living related donor [76] and lower donor–recipient HLA mismatch [7, 77]. The two latter factors suggest that genetic similarities between donors and recipients may be involved in increased recurrence risk [78]. Further research should focus on the assessment of donor and recipient HLA subtypes as well as IgAN risk loci outside the HLA region and to investigate their role in predicting IgAN recurrence. These efforts ultimately may help optimize donor–recipient matching to reduce the risk of IgAN recurrence while improving allograft survival.

Regarding immunosuppressive therapy, while several observational studies support the protective effect of steroid maintenance against IgAN recurrence, the role of other specific induction and maintenance immunosuppressive medications remains controversial. A recent meta-analysis evaluating 20 case–control studies (542 patients with IgAN recurrence and 1385 patients without) found that basiliximab and tacrolimus use were associated with a reduced risk of recurrence [79].

Recent evidence from the TANGO study discovered associations of pre-transplant donor-specific antibodies (DSAs) and post-transplant de novo DSAs with increased IgAN recurrence risk [59].

In addition, several observational studies have identified biochemical predictors of IgAN recurrence, such as higher serum IgA levels, galactose-deficient IgA1, IgA–IgG complexes, IgA–sCD89 complexes, normalized Gd-IgA1-specific autoantibody, APRIL and urine proteins (SERPINA1, transferrin, APOA4 and RBP4) [80–84], although none of these biomarkers have been validated in clinical practice [58].

Proteinuria at the time of recurrence is the strongest negative prognostic factor [59]. In addition, few studies have demonstrated that the presence of multiple MEST‐C components in graft biopsy is associated with a worse prognosis [70, 85].

### Recurrence prevention

Unfortunately, only a few small observational studies have evaluated strategies to prevent IgAN recurrence in KTx recipients.

Tonsillectomy has been shown to be a feasible option in preventing relapsing IgAN in native kidneys [86, 87]. Similarly, it has been shown to decrease proteinuria and induce clinical remission in KTx recipients with IgAN recurrence [88–91]. Accordingly, a recent study conducted in Japan suggested that elective tonsillectomy 1 year after transplant may reduce histological IgAN recurrence, serum Gd-IgA1 levels and mesangial Gd-IgA1 immunoreactivity [92]. Nevertheless, this approach did not significantly improve graft outcomes in European individuals, suggesting ethnic differences in the molecular mechanisms associated with Gd-IgA1 overproduction [93, 94].

Randomized clinical trials will be needed to assess the clinical utility of pre-emptive tonsillectomy.

In clinical practice it is common to avoid ESW due to the potential link with increased IgAN recurrence, as previously reported [95]. Accordingly, a retrospective study has shown that steroid continuation is associated with a lower cumulative risk of graft loss due to IgAN recurrence compared with ESW [96]. Nevertheless, one should be aware of the potential selection bias of these kinds of studies, as patients with ESW more often experience graft dysfunctions that lead to kidney biopsy [39]. Given the lack of strong evidence, future prospective studies are warranted to further address the clinical utility of steroid continuation.

The routine use of protocol biopsies may be considered in patients with IgAN to assess histological forms and early recurrence, although their clinical utility has not been systematically assessed.

### Recurrence management

To date, there are no official guidelines on the treatment of IgAN recurrence, and randomized controlled trials and studies involving large cohorts of KTx recipients are lacking. Consequently, therapy is largely based on the common approach used in native kidneys [97–99].

In KTx recipients, treatment with no further immunosuppressive effects should be considered first to prevent severe infections and oncological risks in this population [100]. The use of renin–angiotensin–aldosterone system (RAAS) blockers may be beneficial to reduce proteinuria and control blood pressure compared with other antihypertensive agents. This may improve early graft prognosis in KTx recipients who require single antihypertensive medication therapy [101]. It should be noted that in the transplant setting, one should be aware of potential significant decreases in estimated glomerular filtration rate (eGFR) and haematocrit with the use of RAAS blockers [102].

Regarding anti-inflammatory therapy, steroids are commonly used in clinical practice, especially in rapidly progressive crescentic forms, although at the cost of increased risk of infection and metabolic side effects associated with high doses [74]. In a retrospective study on patients with biopsy-proven de novo and recurrent IgAN after KTx, Messina *et al.* [103] reported that therapy with pulse and oral steroids for 6 months was associated with improved graft function. Matsukuma *et al.* [104] retrospectively analysed patients with de novo or recurrent IgAN with moderate proteinuria (≥0.5 g/g creatinine) and/or cellular or fibrocellular crescents treated with steroid pulses and found a subsequent effective reduction of proteinuria over 2 years.

Considering other immunosuppressive strategies, mycophenolate mofetil has shown inconsistent results, whereas the utility of RTX and cyclophosphamide was only supported by small cohorts of aggressive IgAN forms [105–107]. For example, in a cohort of 64 KTx recipients with IgAN with endocapillary hypercellularity and proteinuria >1 g/day, RTX in association to standard therapy was associated with a potential favourable effect in the decrease of proteinuria within 12 months and on maintenance of a stable renal allograft function for 3 years [108]. Lopez-Martinez *et al.* [109] used budesonide to treat IgAN recurrence in five KTx recipients, obtaining a significant proteinuria reduction (−26.7%) after 3 months of treatment, but the effect was not confirmed at 6, 12 and 24 months.

**Figure 2:**
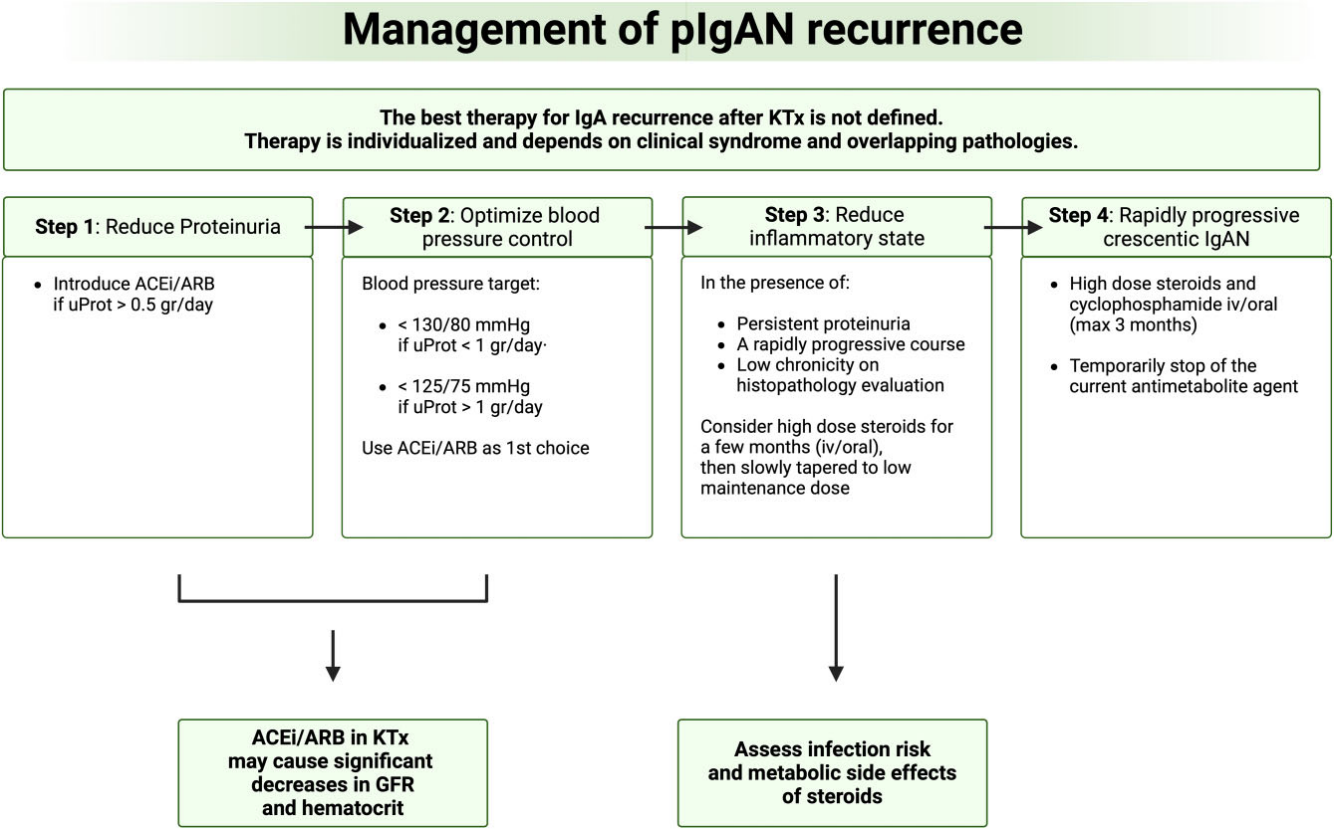
Algorithm for IgAN recurrence in KTx. ACEi/ARB: angiotensin-converting enzyme inhibitor/angiotensin receptors blocker.

An algorithm for managing IgAN recurrence in KTx is proposed in Fig. 2. Even if not studied in KTx recipients to date, a plethora of new potential targeted therapies are under evaluation or recently approved for IgAN treatment in native kidneys, such as drugs targeting the gut mucosal immune system (budesonide), complement system (anti-C5b-9 monoclonal antibodies, C5a receptor blocker), B cells (atacicept and other APRIL and BAFF/APRIL dual inhibitors) and non-immune modulators [sodium–glucose cotransporter-2 (SGLT2) inhibitors, angiotensin II and endothelin 1 antagonists] [97–99]. Future efforts will need to test the safety and efficacy of these promising molecules in KTx recipients. For those acting on the immune system, a strategy to balance infective and oncologic complications should be considered.

## pMN

### Definition, epidemiology and pathogenesis

MN is one of the most common causes of nephrotic syndrome in adults, with a global incidence of 1.2/100 000/year [110]. About 20% of cases are secondary to systemic conditions (infections, autoimmune diseases, cancer or medications); the other cases are considered pMN, mediated by antibodies directed against phospholipase A2 receptor (anti-PLA2R) in 50–80% of cases and against other emerging antigens (i.e. THSD7A, NELL-1 and semaphorin 3B) in the remaining cases [111]. Recent data have shown that pMN has a significant genetic predisposition, which is mainly driven by common variants within the HLA region, the *PLA2R*1 gene and regulators of inflammation such as the *NFKB1* and *IRF4* genes [112]. The most recent GWAS to date has shown that MN is an oligogenic disease, where most of the genetic risk is carried within a few genomic regions with a relatively large effect size. Given the synergistic effect of the HLA and PLA2R1 loci, individuals homozygous for risk variants in both loci have an increased risk of MN with odds ratios of 14–88. A genetic risk score including the top six susceptibility variants was associated with higher proteinuria and anti-PLA2R positivity. In addition, the use of the risk score in combination with the enzyme-linked immunosorbent assay for anti-PLA2R improved the prediction of MN cases [66, 113].

The recurrence rate of pMN after KTx is 10–45% and in up to 70% of cases anti-PLA2R is positive [114]. Histologic recurrence occurs most often during the first year after KTx. Transplant immunosuppression may cause a decrease and disappearance of autoantibodies that may reemerge years after KTx, possibly due to the weaning of immunosuppression (late recurrence) [115].

Grupper *et al.* [116] highlighted some interesting features of pMN recurrence in 21 KTx recipients who underwent follow-up biopsies after pMN recurrence: stage 0 MN at diagnosis had a progressive course in half of cases, spontaneous histological resolution occurred in 33% of cases, post-treatment histological resolution was obtained in <50% of cases and 45% of patients with histological persistence of MN after treatment had clinical remission.

Overall, several studies have shown that recurrent MN is a progressive disease, with a high risk of progression even in the presence of mild proteinuria [115].

### Clinical presentation and histopathology

MN recurrence can be detected at an early stage without any clinical manifestation by protocol biopsies. The first clinical sign of pMN recurrence is proteinuria onset or worsening of previous proteinuria, which should prompt a kidney biopsy. This approach normally detects MN earlier than native kidneys [12].

Histological findings of pMN recurrence are not dissimilar from those in the native forms. Notably, in early cases, subepithelial deposits and basement membrane spikes might not be visible by light microscopy, but only by EM [115]. Similar to native pMN, anti-PLA2R can be detected in the kidney biopsy with immunostaining, and IgG4 is the dominant or codominant immunoglobulin. Notably, C4d staining is often detected with a diffuse granular pattern and C3 staining is not as prevalent in recurrent pMN recurrence as it is in native kidney disease [114].

### Recurrence risk and prognostic factors

The most important pre-KTx risk factors for pMN recurrence are anti-PLA2R positivity, the degree of proteinuria [102] and a prior allograft loss due to recurrent MN. At present, insufficient data are available to understand the relevance to transplantation of the other cited new antigens, which are available in anecdotical reports [117–119].

#### Anti-PLA2R

Given their direct relationship with the risk of recurrence, many studies suggest monitoring anti-PLA2R levels at different times during transplant follow-up [37, 114]. As proposed by Grupper *et al.* [116], positivity of anti-PLA2R testing shortly before or at the time of KTx is strongly associated with disease recurrence (60–76% in anti-PLA2R-positive patients versus 28–30% in anti-PLA2R-negative patients). Moreover, it seems that persistent or re-emerging anti-PLA2R is associated with an increase in proteinuria and a resistant disease [120, 121].

#### HLA polymorphisms

Recently, the role of genetics has been explored for pMN recurrence after KTx [116, 122]. Quintana *et al.* [123] reported the presence of the risk allele HLA DQA1*0501 in six of seven recipients with recurrent MN. Batal *et al.* [124] found that recipient HLA-A3 was associated with pMN recurrence post-KTx in a multivariable model and that HLA-DQ2 and HLA-DR17 antigens were more common in pMN recurrence than in de novo MN. In a small study on 19 KTx recipients with pMN in native kidneys, HLA-DR3 was more frequent in patients with disease recurrence in the graft compared with those without (40% versus 21.4%) [125]. In a work by Berchtold *et al.* [122], donor single-nucleotide polymorphisms in HLA-DRB1 and HLA-DQA1, and three single-nucleotide polymorphisms in the *PLA2R1* gene were associated with post-KTx MN recurrence. This was the first study to test both donor and recipient genetic risk for MN in the transplant setting. The importance of considering donor–recipient HLA characteristics was confirmed also by Chung *et al.* [126], who developed three different predictive models for recurrent MN using the Australian and New Zealand Dialysis and Transplant Registry. Each of these models confirms the predictive relevance of specific recipient and donor HLA antigens. As for other GDs, at present no genomic studies of recurrent MN after transplant exist. Well-designed multicentre studies with adequate power are needed to systematically explore the role of specific immune markers and genetic risk factors in the future.

Concerning prognostic factors after pMN recurrence, data in the literature are scarce. In a recent work, Buxeda *et al.* [127] retrospectively evaluated 71 patients with pMN recurrence after KTx. Recurrence occurred in 25% of patients at a median time of 18 months, without a significant impact on graft survival. Only lower eGFR after recurrence treatment predicted poorer graft survival.

### Recurrence prevention

The KDIGO 2020 guidelines for kidney transplantation suggest anti-PLA2R measurements pre-KTx to inform about the risk of recurrence (evidence 2C) [36].

Furthermore, the KDIGO do not suggest the routine use of RTX or alkylating agents for reducing the risk of recurrence (evidence 2D) [36]. As proposed by Cosio *et al.* [128], pre-emptive use of RTX should be considered in patients with persistently high levels of anti-PLA2R and prior allograft failure caused by recurrent pMN, although the time when prophylaxis should be started is uncertain.

### Recurrence management

Since pMN recurrence has a progressive course in about half of the patients, it is suggested to perform protocol biopsies at different times after KTx to detect and treat it at an early stage. In most of the centres, therapy is started when proteinuria reaches 1 g/day.

RTX leads to complete or partial remission in 70–100% of cases [116]. The optimal dosing is not established. The KDIGO 2021 guidelines for glomerular disease suggest 1 g on day 1 and on day 15 [129], although the dosage should be individualized considering disease severity, treatment response, comorbidities and risk–benefit assessment in recipients with a high risk of infection [125]. Interestingly, the B cell depletion appears to be higher and last longer in KTx than in native kidneys, probably due to the potential additive role of T cell–targeted therapies included in the immunosuppressive regimen for the correct management of transplanted organs. It is common to wait for >1 year to achieve clinical remission [115, 116]. Further studies with increased sample sizes, observational studies and randomized controlled trials are needed for a better evaluation of the efficacy of rituximab in the transplant field.

Some cases do not respond to rituximab, probably because of the existence of long-lived memory plasma cells CD19^−^, CD20^−^ and CD38^+^. Concerning this aspect, using specific therapies against plasma cells has emerged in recent years. Barbari *et al.* [130] reported the first successful case of pMN recurrence treated with bortezomib. In this setting, other therapies such as anti-CD38 monoclonal antibodies might be considered in pMN recurrence. Obinutuzumab, a humanized type II anti-CD20 receptor antibody that induces more profound and long-lasting B lymphocyte depletion than RTX, has been effective in treating refractory pMN in native kidneys and in patients with severe CKD [131]. Its beneficial role in the treatment of pMN recurrence after KTx has been reported in case reports.

Concerning the role of maintenance immunosuppressive therapy in the management of pMN recurrence, interestingly, Buxeda *et al.* [127] showed that spontaneous remission was associated with greater exposure to tacrolimus before recurrence (trough concentration:dose ratio 2.86 versus 1.18; *P* = .028).

**Figure 3:**
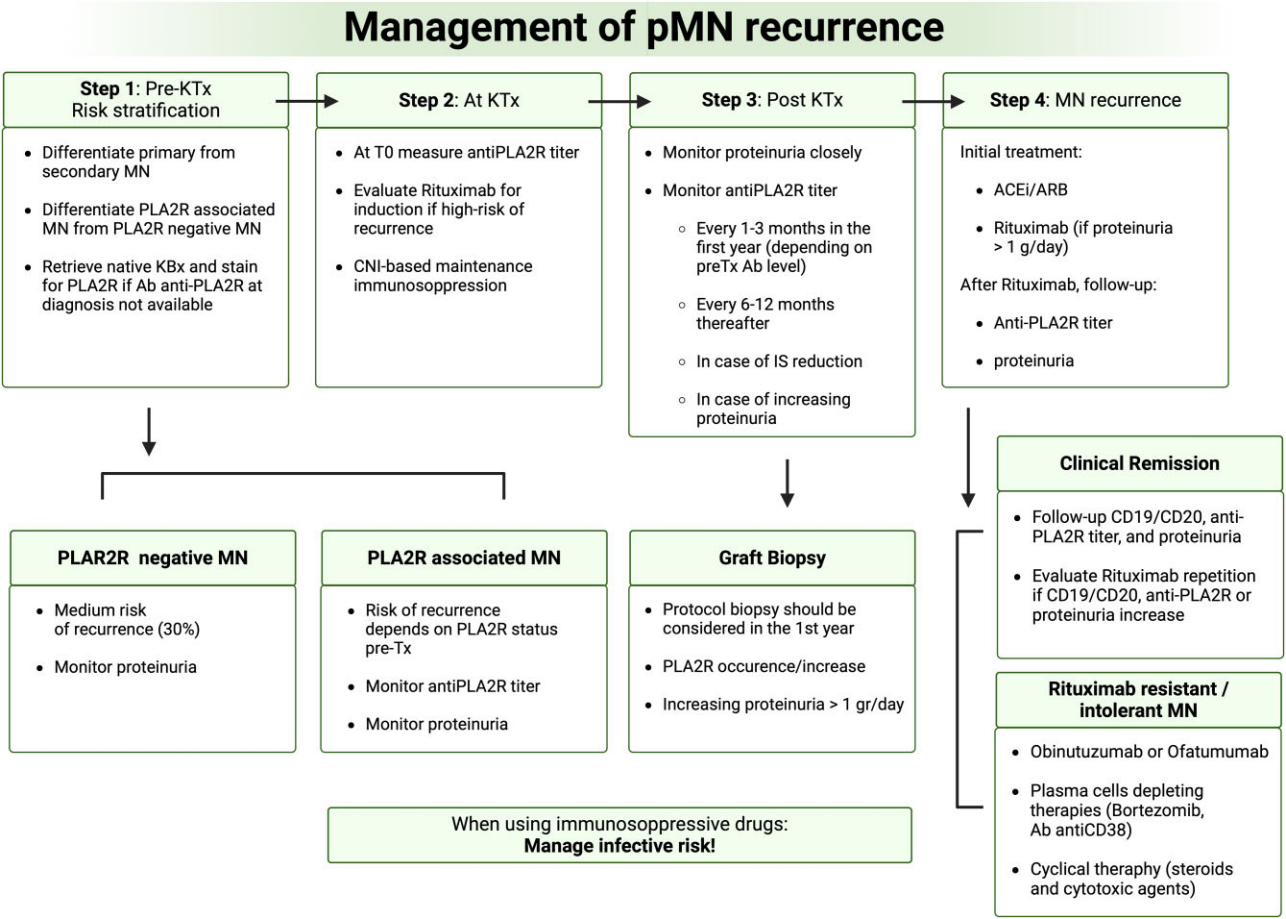
Algorithm for pMN recurrence in KTx. Complete clinical remission is defined as proteinuria <0.3 g/24 h with stable kidney function. Partial remission is defined as a reduction of 50% in baseline proteinuria <3.5 g/24 h with stable kidney function. IS: immunosuppression; ACEi/ARB: angiotensin-converting enzyme inhibitor/angiotensin receptor blocker.

In Fig. 3 we present a clinical algorithm for the management of pMN recurrence. Further research is required to determine whether recurrent pMN forms could benefit from other standard immunosuppressive treatments normally used in native kidneys. In fact, since these patients are usually immunosuppressed with CNIs, the avoidance of alkylating agents such as cyclophosphamide is deemed appropriate to limit severe infective compilations [125]. In this perspective, and also considering recent evidence about the new pathogenetic antigens, future clinical studies should focus on the safety and the possible benefits in the management of immunosuppression with old and new molecules, looking for the best way to limit complications while treating disease recurrence.

## IMMUNE COMPLEX–MEDIATED AND COMPLEMENT-MEDIATED MEMBRANOPROLIFERATIVE GLOMERULONEPHRITIS (MPGN)

### Definition, epidemiology and pathogenesis

MPGN refers to a histological pattern characterized by mesangial hypercellularity, endocapillary proliferation and glomerular basement membrane duplication on light microscopy [132]. According to the new classification proposed by Sethi *et al.* [133], MPGN is now classified as immune complex mediated (IC-MPGN), complement mediated or C3 glomerulopathy (C3G). IC-MPGN can be primary (idiopathic) or secondary to infections, autoimmune diseases or monoclonal gammopathies. C3G is mediated by alternative complement pathway abnormalities and comprises C3 glomerulonephritis (C3GN) and dense deposits disease (DDD) according to EM findings [133].

Unfortunately, only a few and retrospective studies have investigated MPGN recurrence after KTx using the new classification. Among these, Alasfar *et al.* [134] recently studied 34 KTx recipients with ESRD due to MPGN. They found that the recurrence rate of IC-MPGN was 45%, with a median time to recurrence of 8 months. Response to therapy was poor (50% of cases) and in 43% of cases IC-MPGN recurrence caused graft loss, with a median time of 2–18 months after diagnosis. It is known that C3G has a higher recurrence rate after KTx (90% for DDD and 70% for C3GN), with a median time to recurrence of 14–15 months. Overall, graft loss due to its recurrence occurs in up to 50% of patients, with a median time of 18 months after diagnosis, and is more frequent in DDD than C3GN (90% versus 50%) [135, 136].

### Clinical presentation and histopathology

Similar to native kidneys, the clinical presentation of MPGN recurrence can range from urinary abnormalities (microscopic haematuria and/or subnephrotic proteinuria) to nephrotic syndrome to rapidly progressive renal failure. Notably, it is common to detect the recurrence when only the initial urinary abnormalities appear thanks to the usual post-KTx monitoring [6, 137].

From a histological point of view, early MPGN recurrence commonly appears with only mesangial proliferation on kidney biopsy, while advanced MPGN should be differentiated from transplant glomerulopathy [138].

### Recurrence risk and prognostic factors

Mostly monoclonal IC-MPGN recurs, while MPGN with polyclonal IgG deposition, including secondary cryoglobulinaemia, has a lower recurrence risk and a slower progression. Low complement levels, evidence of monoclonal gammopathy and prior graft loss due to recurrence are the main factors related with IC-MPGN recurrence [134].

Risk factors associated with C3G recurrence are young age, heavy proteinuria, crescentic primary disease, prior graft loss due to C3G recurrence and evidence of monoclonal gammopathy [135]. No data exist to support an association between complement testing and recurrent disease after KTx [139].

### Recurrence prevention and management

In case of IC-MPGN, the KDIGO 2020 guidelines suggest investigation for infective, autoimmune or paraprotein-mediated causes prior to KTx to guide treatment and inform about the risk of recurrence (evidence 1C). C3G should be screened for genetic or acquired causes for dysregulation of the complement alternative pathway (evidence 2C). It is suggested, when possible, to treat the underlying cause of IC-MPGN prior to transplantation in an attempt to avoid recurrence (evidence 2C) [36].

Considering C3G, Gonzalez Suarez *et al.* [140] evaluated 12 studies consisting of 122 KTx recipients (73 C3GN and 49 DDD). They found that among non-treated patients there is a high incidence of graft loss (32% in C3GN and 53% in DDD). In treated patients, the lowest incidence of graft loss was noticed in those who received eculizumab instead of PEX or RTX (33%, 42% and 81%, respectively). However, the use of eculizumab remains controversial, but it can be considered for patients at high risk of graft loss, such as those with worsening or high-grade proteinuria and/or progressive decline in kidney function. Plasmatic levels of membrane attack complex C5b9 may help to select good responders to eculizumab [39]. The use of other anti-complement therapies in C3G is based on small open-label trials and case reports with an unknown effect of publication bias [141]. Iptacopan, an oral inhibitor of factor B in the alternative pathway, was associated with a reduction in C3 deposit scores in a phase II study on 11 KTx recipients with C3G recurrence [142]. Pegcetacoplan (a C3 inhibitor) is being studied for assessing safety and efficacy in post-transplant C3G or IC-MPGN recurrence [143].

**Figure 4:**
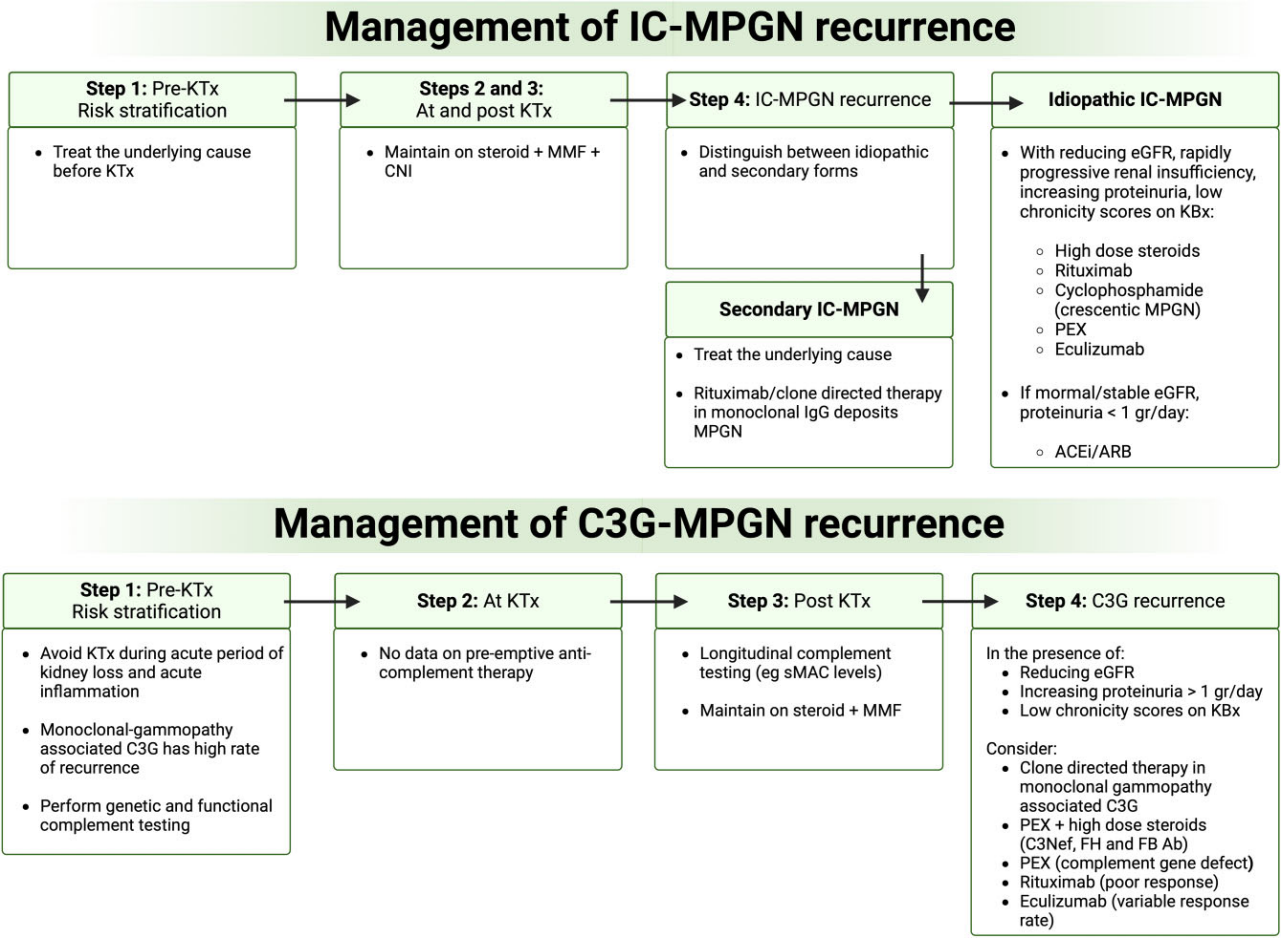
Algorithm for IC-MPGN and CM-MPGN recurrence in KTx. IC: immunocomplex; MMF: mycophenolate mofetil; ACEi/ARB: angiotensin-converting enzyme inhibitor/angiotensin receptor blocker; KBx: kidney biopsy.

In Fig. 4 we propose an algorithm for IC-MPGN and C3G-MPGN recurrence after KTx based on common clinical practice. Given the rarity of these conditions, it is challenging to define precise management guidelines. Future international efforts will be useful to reach higher statistical power and reduce selection bias [39].

## DE NOVO GD

### Definition, epidemiology and pathogenesis

Compared with recurrent pGD, very little is known about the prevalence, pathogenesis, clinical presentation and risk factors of de novo GD. De novo GD is defined as primary (idiopathic) GD occurring in the allograft of KTx recipients who are not affected by the disease in the native kidney.

Estimating the prevalence of de novo GD is quite challenging, given that not all KTx recipients have a diagnosis of their native kidney disease. Indeed, nearly 30% of individuals have an unknown cause of kidney failure, and some of these cases might be undiagnosed GD due to late clinical referral [144]. In addition, on the rare occasion of donor-derived GD, in centres where a kidney biopsy is not performed at the time of transplant, those cases may be misinterpreted as early occurrence of de novo GD.

Despite these challenges, it has been estimated that de novo GDs occur in 5–20% of KTx recipients at 15 years and are associated with a greater risk of subsequent graft loss compared with those without the disease [57, 145–147]. In addition, they typically manifest late during transplantation compared with recurrent disease.

A few retrospective studies have reported that the most common forms of de novo GD are de novo MN, MPGN, IgAN and FSGS in its non-collapsing and collapsing forms [146]. De novo MN, MPGN and IgAN are thought to be the result of immune complex–mediated insults and are thought to involve an autoimmune mechanism [145, 148]. Nevertheless, recent studies have reported associations between forms of de novo GD with acute T cell–mediated and antibody-mediated rejection (AMR), suggesting that a proportion of de novo GD may be mediated by an alloimmune response to donor-derived antigens [149, 150]. For example, Khairallah *et al.* [150] compared 46 KTx recipients with de novo GD with 77 KTx recipients with recurrent GD and demonstrated that de novo GD cases had higher concurrent rates of AMR and a higher proportion of donor-specific antibodies (DSAs) at the time of diagnosis. Among all de novo GDs, the associations with features of acute rejection were highest in de novo MN cases, followed by de novo IgAN and de novo IC-mediated GN not otherwise specified (ICGN-NOS) [78]. In addition, in 28 cases of de novo GN with mesangial immune complex deposits (ICGN-NOS), Giannico *et al.* [149] discovered higher proportions of T cell–mediated rejection compared with KTx without immune complex deposition.

In a small series [146], de novo non-collapsing FSGS has been reported to be the most common form of de novo GD. Nevertheless, de novo FSGS is often associated with several risk factors that induce compensatory hyperfiltration, such as a size discrepancy between recipient and nephron mass, diabetes, hypertension, BK polyomavirus and CNI toxicity. In addition, immune suppressive therapy with sirolimus may induce de novo FSGS due to impairment of podocyte integrity. Therefore, it is likely that de novo non-collapsing FSGS cases are secondary rather than primary.

Given its low incidence, it is difficult to identify the pathogenetic mechanisms of de novo collapsing FSGS. Small studies have reported the occurrence of collapsing FSGS after acute ischaemia in vaso-occlusive disease, as well as sporadic associations with viral infections, such as cytomegalovirus (CMV) and parvovirus B19 [34, 151, 152]. Recent studies have suggested a causative role for the donor *APOL1* risk genotype in the development of collapsing FSGS [153]. In 38 KTx recipients with de novo collapsing FSGS, Santoriello *et al.* [154] reported that *APOL1* high-risk genotypes were independent risk factors for subsequent graft failure.

### Clinical presentation and histopathology

Clinical presentation of de novo GDs can vary from asymptomatic urinary abnormalities, such as haematuria and proteinuria, to overt disease with nephrotic syndrome or renal dysfunction, and therefore it is not substantially dissimilar from the presentation of recurrent GD. Given the chronic immune suppression, de novo GD cases more often present with mild proteinuria or microhaematuria and usually a negative serologic workup [57, 149, 155].

Table 1 shows the clinical and histologic features of de novo GD compared with recurrent GD. De novo MN is the most characterized form of de novo GD and its light microscopy features are similar to native MN [145]. Immunofluorescence studies of post-KTx MN have shown that de novo MN is characterized by dominant or co-dominant IgG1 staining, whereas recurrent MN showed dominant or co-dominant IgG4. In addition, de novo MN cases are more often associated with negative PLA2R staining and negative serum anti-PLA2R, suggesting a different disease mechanism [156, 157]. The other forms of de novo GD are less characterized histologically, and light microscopy does not seem to significantly differ from their recurrent counterparts. EM seems to be an additional important tool to identify potential secondary causes (e.g. viral infections) in KTx recipients with de novo GDs, which is important for prognosis assessment and treatment options [158].

**Table 1:** Main features of recurrent and de novo glomerular diseases after KTx.

Disease	Incidence	Onset time after KTx	Risk factors	Prognostic factors	Outcome
FSGS					
Recurrent	30–50% of patients with pFSGS [13, 159] More often in paediatric patients [159]	2 weeks in children [27] 7.5 months in adults [29]	Prior graft failure due to recurrence [27] Nephrotic syndrome at presentation [13] Rapid progression of FSGS in native kidneys [33] Younger age, White race and living related donor [17, 29] Emerging circulating antibodies (such as anti-nephrin) [160]	Failure to respond to treatment [17, 44, 46, 53]	About 40% of graft loss over a median of 5 years [27]
De novo	Collapsing FSGS occurs in ≈0.6% of renal allografts [34] Not clear for non-collapsing forms [34]	Collapsing forms develop in a mean of 4–5 years after KTx [34] Non- collapsing forms occur >12 months after KTx [34]	Conditions leading to nephron loss and compensatory glomerular hyperfiltration (hypertension, diabetes, rejection, BK polyomavirus) [19, 151, 152, 159] Donor *APOL1* risk genotype for collapsing FSGS [161] CNIs, sirolimus [41]	Proteinuria (including nephrotic syndrome) [31] Hypertension [159] Progressive deterioration of renal function [144]	Graft survival is ≈40% at 5 years after diagnosis [144] Poor outcome when associated with chronic allograft nephropathy [144]
IgAN					
Recurrent	30–40% of patients with IgAN [59]	Not frequent before year 3 after KTx [74] Risk increases with time after KTx [59]	Rapidly progressive course of disease in native kidneys Crescents on the native kidney [74] Male gender [102] ATG-free regimens or ESW [75] Related donors (good HLA match) [79, 102] Serum and urinary biomarkers (i.e. serum IgA levels) [80]	Proteinuria in both native and transplant kidney [84] Crescents at renal biopsy [85]	Graft loss in nearly 44% of cases after years from diagnosis [95]
De novo	Not known, only sporadic cases are reported	Extremely variable, also depending on the renal biopsy strategy of the centre [56, 73]	Not defined	Crescentic forms at renal biopsy have poor outcomes [74, 85]	Not defined
MN					
Recurrent	10–45% of all pMN [115] Up to 70% are anti-PLA2R positive [120]	Most often recurs in the first year after KTx [115] Late recurrence may recur, especially after immunosuppression reduction [116]	Anti-PLA2R positivity before KTx [122] The degree of proteinuria before KTx [115] Some HLA polymorphisms [124, 162]	eGFR after treatment of MN recurrence could predict graft survival [116]	High risk of progression even in the presence of mild proteinuria [114]
De novo	Not clearly defined	Months or years after KTx [115]	Infective or autoimmune triggers (i.e. SARS-CoV-2) [126] Anti-PLA2R levels (even if these forms are often negative) [156]	Proteinuria levels at diagnosis [126] A rapid eGFR decrease [121]	Even more severe than pMN [116]
MPGN					
Recurrent	Recurrence rate for IC-MPGN was 45%. [134] C3G-MPGN has a higher recurrence rate (90% for DDD and 70% for C3GN) [134]	Median time to recurrence of 8 months for IC-MPGN; 14–15 months for C3G-MPGN [136, 138]	IC-MPGN: low complement levels, evidence of monoclonal gammopathy, prior graft loss due to recurrence [137] C3G-MPGN: young age, heavy proteinuria, crescentic primary disease, prior graft loss due to C3G recurrence, evidence of monoclonal gammopathy [137]	No data except for inadequate response to treatment and severe progression [137, 143]	Only 50% of cases respond to treatment, and in 40–50% of cases graft loss occurs 2–18 months after diagnosis for both IC- and C3G-MPGN Graft loss is more frequent in DDD than C3GN (90% versus 50%) [136]
De Novo	Not clearly defined	Seemed to occur later than for recurrent forms, but not clearly defined [138]	Higher proportion of DSAs at time of diagnosis [137, 138] Inadequate therapeutic adherence [143]	The presence of elements of acute rejection in IC-MPGN (both T cell and antibody mediated) [137]	Not defined

### Prognostic factors and management

Given the challenges in the identification of true de novo pGDs and their relatively rare occurrence, it is difficult to identify factors associated with disease progression. All de novo GDs seem to be associated with greater risk of graft loss, especially if there is concurrent extensive interstitial fibrosis/tubular atrophy, lower eGFR and higher proteinuria at the time of diagnosis. Crescentic de novo IgAN is associated with poorer prognosis, similar to recurrent IgAN. De novo collapsing FSGS cases secondary to acute vaso-occlusive disease have a relatively positive outcome if the acute insult is promptly reversed, whereas collapsing FSGS associated with donor *APOL1* risk genotypes tends to have poor outcomes [154].

To date, literature on the treatment of de novo GDs is scarce. Herein, we propose practical guidance for the management and treatment of de novo GDs based on clinical experience.

In all de novo GDs, it is important to identify potential secondary insults, such as features of concomitant acute rejection or viral infections, to guide their management. Blood workup, including a search of DSAs, anti-PLA2R in MN, CMV, Epstein–Barr virus and parvovirus B19 genomes, along with careful examination of transplant histology with the inclusion of EM are important for this purpose.

In general, first-line treatment of de novo GDs includes the use of anti-proteinuric medications (such as RAAS blockers and SGLT2 inhibitors if no contraindications are found) and the removal/treatment of secondary associated insults. Potentiation of immunosuppressive therapy, especially in the presence of overt nephrotic syndrome, crescents and/or histologic features of rejection may be considered, although there are currently insufficient data to guide management of these diseases.

## CONCLUSIONS

In conclusion, the current literature has shown that pGD recurrence and de novo GDs are important causes of allograft failure.

To reduce the negative impact of recurrent and de novo GDs on graft survival, we underline the following points to be implemented in clinical practice:

• Identification of the original cause of kidney failure before KTx
• Adequate information to patients about the risk of recurrence
• Selection of appropriate pre-emptive therapy at KTx able to reduce disease recurrence in high-risk patients
• Monitoring patient adherence to immunosuppressive maintenance therapy
• Establishment of predictive markers of recurrence after KTx
• Appropriate monitoring strategies to detect recurrent and de novo GDs at an early stage
• Identification of appropriate therapies for recurrent and de novo GDs
• Balance of benefits and risks of additional immunosuppressive therapies.

## Data Availability

No new data were generated or analysed in support of this research.
